# Changes in Tongue Area, Pharyngeal Area, and Pharyngeal Airway Velocity after Correction of Mandibular Prognathism

**DOI:** 10.3390/jcm10194560

**Published:** 2021-09-30

**Authors:** Chun-Ming Chen, Ting-Ying Yu, Szu-Ting Chou, Jung-Hsuan Cheng, Shih-Chieh Chen, Chin-Yun Pan, Yu-Chuan Tseng

**Affiliations:** 1 Department of Oral and Maxillofacial Surgery, Kaohsiung Medical University Hospital, Kaohsiung 80756, Taiwan; komschen@gmail.com; 2 School of Dentistry, College of Dental Medicine, Kaohsiung Medical University, Kaohsiung 80756, Taiwan; cherubiz01@gmail.com; 3 School of Electrical and Computer Engineering, Georgia Institute of Technology, Atlanta, GA 30332, USA; tyu304@gatech.edu; 4 Department of Orthodontics, Kaohsiung Medical University Hospital, Kaohsiung 80756, Taiwan; zinglontion@hotmail.com (J.-H.C.); anakin0430@gmail.com (S.-C.C.); spig.pan6363@gmail.com (C.-Y.P.)

**Keywords:** pharyngeal airway area, tongue area, airway velocity, mandibular prognathism, mandibular setback, sleep apnea, horizontal setback of menton, vertical change of menton

## Abstract

This study aimed to investigate the correlation between the amount of mandibular setback, and the related changes of the tongue area, pharyngeal area, and pharyngeal airflow velocity. Twenty-five patients treated for mandibular prognathism, and serial cephalograms were obtained (T1: preoperation, T2: more than one year postoperation). The postoperative area of the tongue, pharyngeal airway space, and pharyngeal airflow velocity were investigated. Statistical analysis was performed with the Student *t*-test and Pearson correlation. The amount of mandible setback was significant after surgery (12.8 mm; *p* < 0.001). The pharyngeal area was significantly reduced 115.5 mm^2^ (*p* = 0.046). There was a slight reduction of the tongue area (43.2 mm^2^; *p* = 0.305) and an increase of pharyngeal airflow velocity (0.3 m/s; *p* = 0.133). The Pearson correlation coefficient test showed no statistical significance among the amount of horizontal setback and vertical movement of the mandible, such as the reductions in the tongue area, the pharyngeal airway space, and the increase in pharyngeal airflow velocity. Larger amounts of mandibular setback caused a significant reduction of pharyngeal airway area, but without significant changes of the tongue area and pharyngeal airflow velocity.

## 1. Introduction

The pharynx, which is part of the upper airway, is divided into three regions according to location: the nasopharynx, the oropharynx, and the laryngopharynx or hypopharynx. The pharynx’s primary functions are swallowing and respiration as the passageway of air, food, and fluids. Located at the floor of the mouth and the inner side of the mandible, the tongue is a major sensory organ that also plays a crucial role in speech production. The tongue is arguably the organ with the most active function in the oropharyngeal system since it participates in the physiological activities of chewing and swallowing [1,2]. However, its position affects the size of the airway space. It is well known that the location of the pharynx and tongue could affect the pharyngeal airflow.

Therefore, the pharyngeal airflow and the change in airflow pattern during respiration are crucial to physiological functions, such as pulmonary ventilation [3,4].

**Figure 1:**
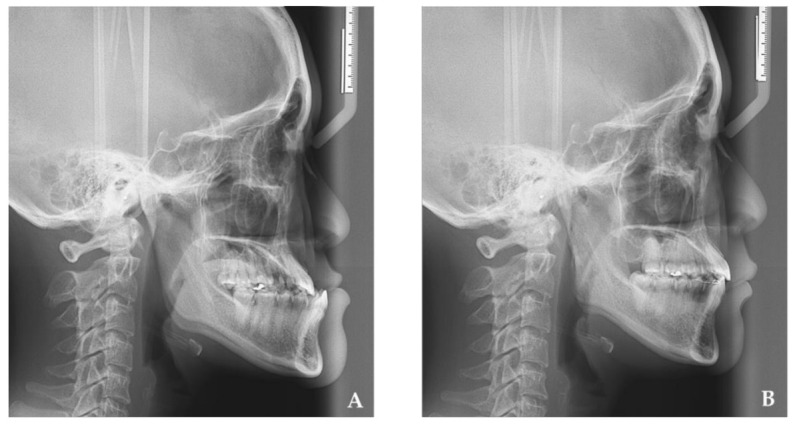
The changes of pharyngeal airway space after mandibular setback by intraoral vertical ramus osteotomy: (**A**) preoperation cephalogram, (**B**) postoperation cephalogram.

Mandibular prognathism was treated with mandibular setback, which achieves adequate occlusion and improves masticatory and swallowing functions. In the literature [5,6,7,8,9], orthognathic surgeries have been confirmed to contribute to changing the shape of the pharynx. (Figure 1).

Aboudara et al. [10] compared imaging information about nasopharyngeal airway size between a lateral cephalometric headfilm and a 3-dimensional cone-beam computed tomography scan in adolescent subjects. Moderately high (*r* = 0.75) correlation was found between airway area and volume; the larger the area, the larger the volume. Bronoosh and Khojastepour [11] investigated pharyngeal airways using lateral cephalogram vs. CBCT Images in a cross-sectional study. They found a strong correlation (*r* = 0.831) between lateral cephalogram (pharyngeal area, mm^2^) and CBCT (pharyngeal volume, mm^3^) measurements of pharyngeal airways. Therefore, the 2D pharyngeal area could be a model in the relation of 3D pharyngeal volume. Masoumi et al. [12] performed a 2-dimensional MRI images model of the ventricular system for the computational fluid dynamic analysis. The cerebrospinal fluid (CSF) pressure and flow velocity in different areas were investigated. They found that 2-dimensional models could provide a quantitative simulation of CSF flow in the ventricles. Na et al. [13] performed computational analysis of airflow dynamics for predicting collapsible sites in the upper airways. They reported the contour of flow velocity and pressure on the midsagittal plane in the upper airways of the retruding and protruding jaws.

Obstructive sleep apnea (OSA) is a condition in which breathing presents intermittent and repetitive stop due to pharyngeal airway collapse during sleep. The collapse of the pharyngeal airway leads to hypoxemia and results in detrimental effects on general health. Considering the mandibular setback operation, the narrowing of pharyngeal airway space has been implicated in the development of OSA. The screening tools for OSA risk assessment in both subjective and objective observations include questionnaire, Mallampati scores, pharyngeal airway volume, pulse oximetry, polysomnography, and apnea–hypopnea index (AHI), etc. Dalewski et al. [14] reported that the compositions of modified Mallampati scores, upper airway volume measurements, and the Berlin questionnaire are useful and reliable to assess the risk of snoring. Sata et al. [15] investigated the polysomnographic study in AHI over 100 and showed extremely high frequency of apneas rather than hypopneas. Sata et al. [15] also found that the craniofacial structures (cephalometric findings) of OSA patients with AHI over 100 were not significantly different from those of OSA patients with AHI between 15 and 90. They suggested that the upper airway may more easily collapse during sleep by factors other than the upper airway anatomical morphology in patients with AHI over 100 than in patients with AHI between 15 and 90. Song et al. [16] used computational fluid dynamics simulation of changes in the morphology and airflow dynamics of the upper airways in OSAHS patients after treatment with oral appliances. They reported the change tendencies of airflow velocity in that of palatopharynx and glossopharynx decreased significantly (*p* < 0.05) in the mid-sagittal plane of the upper airway, from 11.55 m/s to 8.81 m/s post-treatment, representing a decline of 23.7%.

Moreover, Greco et al. [17] reported that pharyngeal airway space was clearly narrowed after mandibular setback by performing the cephalometric analysis. Chen et al. [18] found that tongue length and oropharyngeal airway space were significantly decreased after mandibular setback surgery. This indicates the changes in the airway space and the position of the tongue are the critical factors for the maintenance of pharyngeal airway after mandibular setback. This study investigated the effect of mandibular surgery on the tongue area in patients treated with mandibular setback. First, the null hypothesis of the present study was that the amounts of mandibular setback were not significantly correlated with the changes of the tongue area. Our study further explored the change of the postoperative tongue area and how it affects the pharyngeal airway and pharyngeal airflow velocity for the maintenance of respiration function. Second, the null hypothesis of the present study was that the amounts of mandibular setback were not significantly correlated with the pharyngeal area and pharyngeal airflow velocity. We expect that there is no significant difference between pre- and post-pharyngeal airflow velocity.

## 2. Materials and Methods

The participants were 25 patients with mandibular prognathism. All subjects needed to meet these eligibility criteria: (1) had Class III malocclusion caused by mandibular prognathism; (2) no history of facial bone injury or other congenital craniofacial anomalies; (3) only received bilateral intraoral vertical ramus osteotomy (IVRO) without genioplasty; (4) surgeries were performed by the same surgeon; (5) had no sleep-related breathing disorder; and (6) obesity patients (BMI more than 30) were excluded. Obesity is characterized to be an important rick factor of OSA. Prior to operation, deep breath exercise was recommended to patients in order to train their breathing muscles and increase lung capacity during the maxillomandibular fixation period. The pre- and postoperation crainocervial (C4SN) angle was recorded.

**Figure 2:**
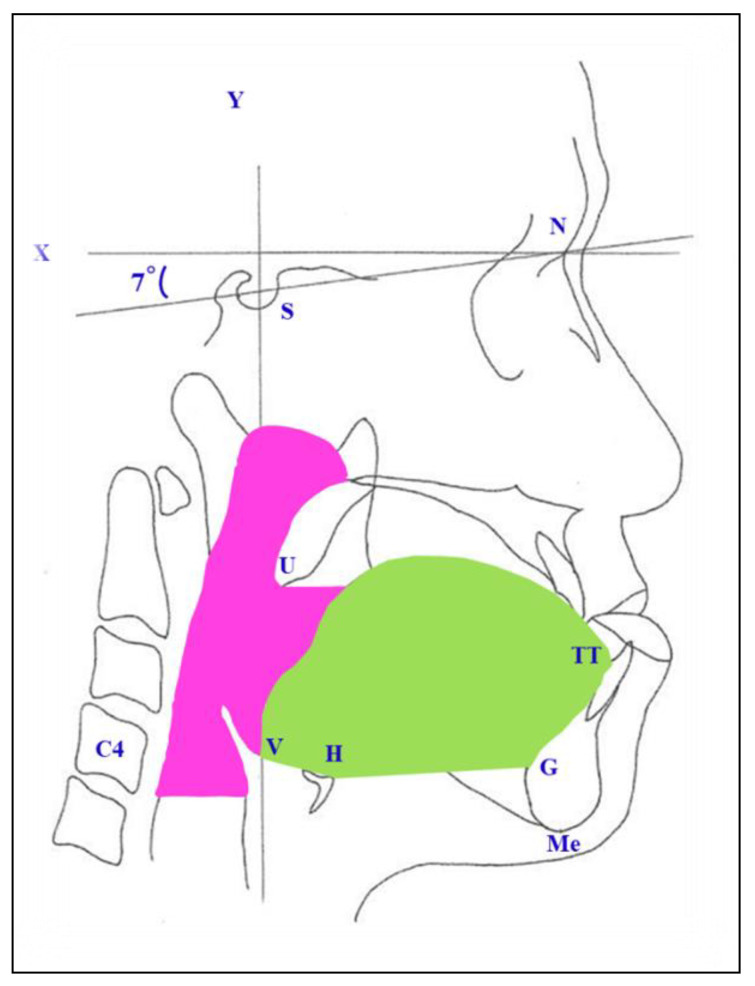
Reference points: sella (S), nasion (N), menton (Me), tongue tip (TT), the most prominent point of the mandibular symphyseal posterior border (G), vallecula epiglottica (V), hyoid bone (H), uvula (U), fourth cervical vertebra (C4). *x*-axis: Constructed by drawing a line through N 7° up from SN line. *y*-axis: a line through S perpendicular to the *x*-axis. Pharyngeal area (pink color): lower border horizontal plane through the inferoanterior point of C4. Tongue area (green color).

**Figure 3:**
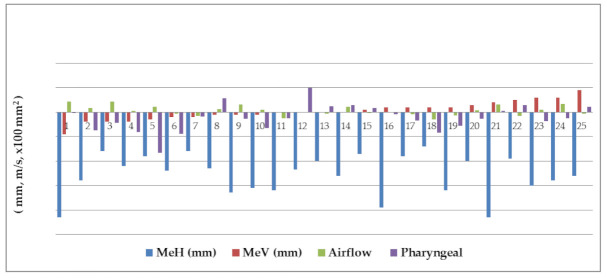
The summary of patients (n = 25) at the final surgical changes (T12).

Lateral cephalograms were taken before surgery (T1) and more than 1 year after surgery (T2) (Figure 1). Reference points, as well as the areas of the tongue and the pharyngeal airway space, were set as follows in Figure 2, S, sella; N, nasion; Me, menton; H, the most superior and anterior point of hyoid bone; G, the most prominent point of the mandibular symphyseal posterior border; V, vallecula epiglottica; TT, tongue tip; ANS, anterior nasal spine; PNS, posterior nasal spine; C4: inferoanterior point on the fourth cervical vertebra. The research defined the three reference lines as: (1) the S-N line; (2) the *x*-axis: passing over the S-N line at 7° and through the N; and (3) the *y*-axis: a line vertically passing through the *x*-axis and penetrating the S. The changes in Me setback, the tongue area, pharyngeal airway space, and pharyngeal airflow velocity were measured from preoperation and postoperation cephalograms. The directions of movement were defined in the horizontal (+: forward; −: backward) and vertical (+: downward; −: upward) directions. (Figure 3) Concerning the reliability and reproducibility of measurements, the methods of Albarakati et al. [19] and Miao et al. [20] were performed. Twenty-five cephalograms were randomly measured twice in a 7-day interval. Systematic errors were evaluated using a paired 𝑡-test for Me, and no significant difference (*p* = 0.6116) was observed. Accidental errors were calculated using the Dahlberg formula, which is expressed as follows: accidental errors = √Σ𝑑^2^/2𝑛, where 𝑑 represents the difference between the two sets of data and 𝑛 represents the number of measurements. Accidental errors (0.44 mm on average) were less than 0.5 mm, thus indicating the accuracy of the measurements and confirming the consistency in our study.

**Figure 4:**
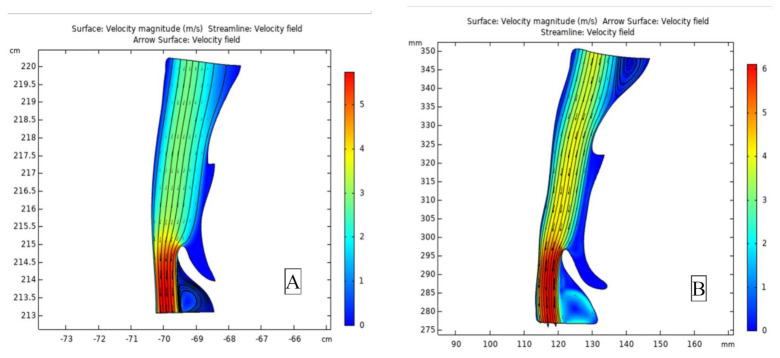
(**A**) Preoperation airflow velocity, (**B**) Postoperation airflow velocity.

Airflow velocity was calculated using COMSOL Multiphysics^®^ 5.5 software (COMSOL, Inc., Burlington, MA, USA). The image files were first converted into DXF files before software processing. By inputting the material properties of air, setting up the computational fluid dynamics parameters (inlet and outlet), and building up grids for meshing, the airflow velocity and its vector plot can be obtained. (Figure 4) Finally, this research utilized the Student’s *t*-test and Pearson’s chi-square test to investigate the differences between the preoperation and postoperation data. Regarding absolute correlation coefficient values, 0–0.19 was considered very weak, 0.2–0.39 was considered weak, 0.40–0.59 was considered moderate, 0.6–0.79 was considered strong, and 0.8–1 was considered very strong. The statistical significance was set at *p* < 0.05.

## 3. Results

The pre- and postoperation C4SN angle were 100.4 and 102.5 degrees, respectively. As shown in Table 1, regarding the change in Me position 1 year after surgery (T12), the mean horizontal setback was 12.8 mm (*p* < 0.001). A considerable difference between preoperation and postoperation location was observed, indicating a notable amount of mandibular setback. However, the difference in vertical movement, which was downward by an average of 0.2 mm (*p* = 0.577) is insignificant. The clockwise rotation (CR) and counterclockwise rotation (CCR) of the mandible (Me) were summarized in Table 1. The horizontal mandibular setback (Me) presented the significant difference between preoperation and postoperation in the CR (13.0 mm), CR (12.7 mm), and no rotation (12.7 mm). The vertical change of (Me) presented the significant difference between preoperation and postoperation in the CR (1.9 mm) and CR (−1.6 mm).

As shown in Table 2, the reduction in the tongue area from before and 1 year after surgery was 43.2 mm^2^, but this difference is relatively small (*p* = 0.305). This suggests that although the magnitude of postsurgical Me was considerable, the tongue area was only slightly compressed. However, the pharyngeal airway space decreased by an average of 115.5 mm^2^ from before to 1 year after surgery, a significant difference (*p* = 0.046). In other words, when the rotation direction of Me was substantial in surgery, the pharyngeal airway space was clearly compressed. Although the pharyngeal airway space was reduced significantly, the postsurgical increase in pharyngeal airflow velocity of 0.3 m/s was trivial (*p* = 0.133). The CR group and no rotation group revealed no significant difference between preoperation and postoperation in the airflow velocity, pharyngeal airway area, and tongue area. The CCR group presented the significant difference between preoperation and postoperation in the airflow velocity (+ 0.8 m/s) and pharyngeal airway area (−255.5 mm^2^).

As shown in Table 3, the Pearson’s test revealed a very weak correlation between the changes of the vertical position of Me and horizontal position of Me after surgery (*r* = 0.029). Similarly, there is no significant correlation between horizontal changes of Me and the reduction in the pharyngeal airway area (*r* = −0.127, *p* = 0.545) and tongue area (*r* = 0.018, *p* = 0.932). Therefore, the first null hypothesis was accepted. Moreover, the horizontal changes of Me associated with the changes of pharyngeal airflow velocity (*r =* −0.384, *p* = 0.058) were weak. The Pearson’s test demonstrated no significant correlations between the changes in vertical retraction, the reduction in the pharyngeal airway area and tongue area, or the increase in the airflow velocity. Furthermore, the increasing airflow velocity is hard to relate with the decreased area of the tongue and pharyngeal airway. The secondary null hypothesis in our study was accepted; the results met with our expectations.

## 4. Discussion

Several studies explored the prevalence of Class I, II, and III malocclusion in different ethnic groups. Edward Angle found that 69%, 23%, and 3.4% of the surveyed white individuals were classified as Angle Class I, Class II, and Class III malocclusion, respectively [21]. A study by Altemus [22] reported that 83%, 12%, and 5% of black individuals in the United States aged between 12 and 16 years had Angle Class I, II, and III malocclusion. Silva and Kang [23] presented that 69.4%, 21.5%, and 9.1% of Latino Americans aged between 12 and 18 years were found to have Class I, II, and III malocclusion. Tang [24] reported the prevalence of malocclusion and treatment need in young Chinese adults. The most commonly occurring feature was Class I (63.9%), followed by Class II (21.3%), and Class III malocclusion (14.8%). Therefore, the prevalence of Angle Class III occlusion in Chinese is higher than that among white, black, or Latino individuals.

Mandibular prognathism is classified as severe Angle Class III malocclusion. In families with mandibular prognathism, parents and children often have similar physiognomic characteristics. Studies by Hunter [25] and Nakasima [26] had revealed a considerable direct correlation between parents’ and children’s facial bone dimensions. This discovery indicates that Angle Class III malocclusion is hereditary. Studies [5,17] on patients with mandibular prognathism undergoing mandibular setback have reported that mandibular backward movement also resulted in the retraction of the tongue, the hyoid bone, and the position of the epiglottis. These movements resulted in the narrowing of the pharyngeal airway space after orthognathic surgery. Riley et al. [27] indicated that orthognathic surgery for mandibular setback gave rise to obstructive sleep apnea afterward since it caused the narrowing of the airway. The anatomical positions of the mandible, the tongue, and the pharyngeal airway are closely related. Any severe reduction in the tongue area and pharyngeal airway space might cause sleep apnea. Thus, surgeons must clearly understand all changes in these relevant structures to prevent any sleep disorder from occurring when a patient’s degree of mandibular setback is extremely high. It is necessary to keep track of the shift in these areas after surgery. Hence, plans for surgical treatment for patients with facial deformities should not only evaluate the changes in the tongue and pharyngeal airway space but also take related airflow in the pharyngeal airway into consideration.

Turnbull and Battagel [28] reported that the mandible was a setback with 5.8 mm and that 6 weeks later, tongue thickness had increased by 1 mm. Gokce [29] performed the mandibular setback by an average of 6.5 mm, and the tongue thickness 1 year after surgery decreased by 0.06 mm. Each piece of research shows little difference in tongue thickness. Compared to previous reports [28,29], the degree of mandibular setback (more than twofold) was considerably greater in the present study. However, this led to only a minimal reduction in the tongue area (1.4%); no significant differences in the degree of setback were observed. In other words, the increase in tongue thickness (observed immediately after surgery) is caused by the compression of the tongue retraction. Over the course of 1 year, the tongue adapted to the postsurgical structure to maintain regular respiration and thereby the stability of the tongue area.

In the mandibular setback, muscle and soft tissue attached to the inner side of the mandible retracted the circular formations of soft tissue. That led upward to the palatine uvula, and simultaneously drew back the tongue. As a result, the pharyngeal airway space was compressed. According to Muto et al. [6], mandibular setbacks led to average reductions of approximately 2.6 and 4.0 mm in the pharyngeal airway space and the posterior part of the palate, respectively. In a study by Tselnik and Pogrel [7], an average mandibular setback of 9.7 mm led to an approximately 12.8% decrease in the pharyngeal airway space. These present research findings are consistent with previous studies: the pharyngeal airway space reduced drastically (around 7.1%) 1 year after surgery. This indicates that the pharyngeal airway space was compressed when the degree of mandibular setback was considerable. Moreover, greater pharyngeal airway area compression and airflow velocity were observed in CCR group.

In such cases (severe mandibular setback) immediately after surgery, respiratory smoothness is constrained by and relevant to oxygen supply. The difficulty of breathing could result in dyspnea and lead to apnea. For monitoring postoperative patients’ oxygenation, our patients were monitored with continuous pulse oximetry during sleep. In this study, no abnormal respiration was observed in the patients immediately after surgery. According to our prior research [18], smoother airflow will result in patients by slightly raising the head after mandibular setback surgery. It is confirmed that increasing the craniocervical (C4SN) angle could alleviate the inconvenience caused by the reduced airway space. This indicates that mandibular setbacks, pharyngeal airway space, and head position are highly correlated.

Sleep apnea is a disorder in which the collapse of soft tissue in the airway during sleep leads to airflow obstruction, hindering the normal entry of air into the lungs and thereby causing a sharp drop in blood oxygen levels. A treatment for sleep apnea involves the use of a continuous positive airway pressure machine, which provides mechanical ventilation through a mask covering the nose and mouth. Pressurized air is directly applied to the upper airway, preventing airway collapse or obstruction through the maintenance of steady airflow, thereby improving respiratory rate as well as sleep duration and quality. Mandibular setback causes the compression of the pharyngeal airway space, thus increasing airflow velocity, which reaches its peak value in the narrow oropharyngeal area. [30,31] In the present study, although the procedure achieved considerable mandibular setback in all the patients, the increase in the airflow velocity (12%) was not noticeable.

In addition, in our prior research [32], the pharyngeal airway dimensions of patients with mandibular prognathism were much larger than those of patients with skeletal Class I or Class II relation. Although the airflow velocity increased after mandibular setback surgery shrunk the pharyngeal airway region, it remained in the normal range and was comparable to that of those of skeletal Class I or Class II patients. Indeed, our patients did not detect any respiratory distress or feel any discomfort. There are some limitations in this study. First, sample size was only 25 patients and it was not evident enough to provide clinical consideration. Another limitation was the study conducted two-dimensional (2D) analysis. Further research should perform the 3D images for evaluating the change of pharyngeal airway volume and airflow velocity.

## 5. Conclusions

Following the mandibular setback surgery, reduction in the total pharyngeal airway area was significant. Reduction in the total tongue area and increment in the pharyngeal airflow velocity were non-significant. This result met with our expectation, there is no significant difference between pharyngeal airflow velocity pre- and post-surgery in mandibular prognathism patients treated with mandibular setback.

## Figures and Tables

**Table 1:** The clockwise (n = 11), counterclockwise (n = 10), and no rotation (n = 4) of menton (Me) at the final surgical change (T12) in the Student’s *t*-test.

Me (mm)		Mean	SD	*p* Value	Significant
Horizontal change					
Clockwise Rotation		−13.0	4.71	<0.001	*
Counterclockwise Rotation		−12.7	4.27	<0.001	*
No Rotation		−12.7	2.53	0.002	*
Total		−12.8	4.03	<0.001	*
Vertical change					
Clockwise Rotation		1.9	1.22	<0.001	*
Counterclockwise Rotation		−1.6	1.21	0.003	*
No Rotation		0.0	0.00	0.364	NS
Total		0.2	1.91	0.578	NS

*n*: number of patient. *: Significant *p* < 0.05; NS: Not significant.

**Table 2:** The clockwise (n = 11), counterclockwise (n = 10), and no rotation (n = 4) of the tongue area and pharyngeal airway area at the final surgical change (T12) in the Student’s *t*-test.

Variables		Mean	SD	*p* Value	Significant
Airflow velocity (m/s)					
Clockwise Rotation		0.0	0.96	0.939	NS
Counterclockwise Rotation		0.8	0.98	0.026	*
No Rotation		−0.1	0.93	0.780	NS
Total		0.3	1.01	0.133	NS
Pharyngeal airway area (mm^2^)					
Clockwise Rotation		−88.4	174.38	0.124	NS
Counterclockwise Rotation		−255.5	297.96	0.024	*
No Rotation		160.4	254.71	0.297	NS
Total		−115.5	274.34	0.046	*
Tongue area (mm^2^)					
Clockwise Rotation		5.1	277.97	0.953	NS
Counterclockwise Rotation		−88.1	133.13	0.066	NS
No Rotation		−63.4	113.19	0.344	NS
Total		−43.2	205.96	0.305	NS

*n*: number of patients. *: Significant *p* < 0.05; NS: Not significant.

**Table 3:** Pearson test of final surgical change (MeT21H and MeT21V) among the pharyngeal airway area, tongue area, and airflow velocity.

Variable	MeT21H		MeT21V		Tongue Area		Pharyngeal Area		Airflow Velocity	
	*r*	*p* Value	*r*	*p* Value	*r*	*p* Value	*r*	*p* Value	*r*	*p* Value
MeT21H	1		0.029	0.891	−0.127	0.545	0.018	0.932	−0.384	0.058
MeT21V	0.029	0.891	1		0.273	0.186	0.364	0.074	−0.329	0.108
Pharyngeal area	−0.127	0.545	0.273	0.186	1		0.106	0.614	−0.041	0.846
Tongue area	0.018	0.932	0.364	0.074	0.106	0.614	1		−0.201	0.335
Airflow velocity	−0.384	0.058	−0.329	0.108	−0.041	0.846	−0.201	0.335	1	

*r*: Correlation coefficient significant, *p* < 0.05.

## Data Availability

The data used to support the findings of this study are included within the article. The data used to support the findings of this study are available from the corresponding author upon request.
